# Clinical considerations for the development of biosimilars in oncology

**DOI:** 10.1080/19420862.2015.1008346

**Published:** 2015-01-26

**Authors:** Mark A Socinski, Giuseppe Curigliano, Ira Jacobs, Barry Gumbiner, Judith MacDonald, Dolca Thomas

**Affiliations:** 1 University of Pittsburgh; Pittsburgh, PA USA; 2 Division of Experimental Therapeutics; European Institute of Oncology; Milan, Italy; 3 Pfizer Inc.; New York, NY USA; 4 Pfizer Inc.; San Diego, CA USA; 5 Pfizer Limited; Walton Oaks, Surrey, UK

**Keywords:** biosimilar, biologic therapy, clinical, monoclonal antibody, regulatory

## Abstract

Despite availability of biologic therapies, limited patient access to many of the most-effective cancer treatments affects overall health outcomes. To address this issue, many governments have enacted legislation for the approval of biosimilars. The term “biosimilar” refers to a biologic product that is developed to be highly similar, as opposed to identical, to a licensed biologic product (the reference or innovator product), such that, per US Food and Drug administration draft guidelines, “no clinically meaningful differences [exist] between the biological product and the reference product in terms of safety, purity, and potency.” This article presents some considerations about the development of biosimilars in cancer treatment through an overview of biosimilars from a clinical perspective. Topics covered include the development requirements and unique regulatory requirements for biosimilars, labeling considerations, potential limitations to the uptake of biosimilars, and review of some biosimilars in development for oncology indications.

## Abbreviations

CI: confidence interval
EBE: European Biopharmaceutical Enterprises
EMA: European Medicines Agency
EPAR: European Public Assessment Report
FDA: US Food and Drug Administration
HER2: human epidermal growth factor receptor 2
MBC: metastatic breast cancer
ORR: overall response rate
PD: pharmacodynamics
PK: pharmacokinetics
SmPC: summary of product characteristics
WHO: World Health Organization

## Introduction

Cancer is the most economically devastating cause of death worldwide.^1^ The World Health Organization (WHO) estimates that the medical cost associated with treating new cancers in 2010 was $163 billion (calculated in US dollars).^2^ These estimates do not include the economic impact associated with premature death and disability or the costs of time by caregivers or transportation to treatment facilities, which increase the total annual estimated cost of treating newly diagnosed cancers to US$310 billion in 2010.^2^ These costs are expected to continue to increase. For example, in the United States alone the annual cost of cancer care for all cancers (not just newly diagnosed cancers) is expected to increase dramatically from an estimated $125 billion in 2010 to a projected $173 billion in 2020.^3^

Despite regulatory approval of a number of biologic agents in the treatment of various cancers, patient access to treatment is often limited.^4,5^ Some healthcare systems ration high-cost treatments by limiting the number of treatments or restricting use to specific patient populations even though this might not be the most cost-effective strategy.^6^ As a result, these systems deny access to many patients who could be helped.^6^ To date, worldwide access to highly effective cancer treatments remains an unmet medical need in many countries.

Many governments worldwide have enacted legislation to allow regulatory approval of biosimilars.^7-10^ The term “biosimilar” refers to a biologic product that is developed to be highly similar, as opposed to identical, to an existing licensed biologic product (i.e., the reference or innovator product), such that there are “no clinically meaningful differences between the biological product and the reference product in terms of safety, purity, and potency of the product.”^9^ Because these reference products are typically large, structurally complex proteins, even minor changes in the manufacturing process can produce post-translational structural differences that can affect the safety and potency of the product.^9,11^ As a result, biosimilars cannot be considered generic equivalents to the reference product.^9,10^

Here, we present an overview of biosimilars from a clinical perspective. We discuss the unique developmental processes used for biosimilars, the specific regulatory requirements, labeling considerations (including how labeling may affect clinicians), and potential limitations to the uptake of biosimilars in clinical use. We also provide information about some biosimilars in development for oncology indications.

## Developmental and Regulatory Processes for Biosimilars

A critical challenge faced by biosimilar developers is that the manufacturing process for the reference product is proprietary. As a result, the biosimilar developer must analyze the reference product extensively and use a process of reverse engineering to produce a biologic agent that is highly similar to the reference product. This requires substantial knowledge and expertise regarding the development and manufacture of biologics.

Because biologics are relatively large and complex proteins that are difficult to characterize, the regulatory process for biosimilar approval is not the same as that used for small-molecule generics. As a result of the unique considerations related to biosimilars, regulatory agencies have established guidelines specifically for their approval. Regulatory requirements for approval of biosimilars are generally consistent across guidelines of the European Medicines Agency (EMA), Health Canada, WHO, and the draft guidelines from the US Food and Drug Administration (FDA). Although minor differences exist among the agencies (**Table 1**), all require a stepwise approach that includes analytical studies, at least one human pharmacokinetic (PK) study, and generally a minimum of one efficacy and safety study intended to support a demonstration of biosimilarity.^7-10^ The size and scope of the clinical program depend on the degree of similarity to the reference product demonstrated in non-clinical development, i.e., through comparative analytical and non-clinical data (in vitro or in vivo). Ultimately, regulatory decisions for approval are based on the “*totality of the evidence*” in support of the biosimilar in comparison to the reference product.^7-10^

**Table 1.** Comparisons of regulatory requirements for biosimilars

	EMA Guidelines^7^	FDA Draft Guidance^9^	Health Canada Guidance^8^	WHO Guidelines^10^
Non-clinical in vitro studies	Target binding; signal transduction, functional activity/viability of cells of relevance	Structural analyses, functional assays	Receptor-binding or cell-based assays	Receptor-binding or cell-based assays
Non-clinical in vivo studies	If in vitro comparability is identified as satisfactory, without factors that would block direct entry to humans, animal studies may not be required (risk-based approach)	Unless determined not necessary by FDA, includes animal toxicity assessments, animal PK and PD measures, animal immunogenicity	PD studies relevant to clinical application, toxicity (including toxicokinetic parameters), and other relevant safety observations	Relevant biologic/PD activity, toxicity
Clinical studies	Comparability demonstrated in stepwise process using PK (and PD if feasible), followed by clinical efficacy and safety trials	Study or studies including assessments of immunogenicity and PK or PD to demonstrate safety, purity, potency	PK, PD, efficacy, safety, including immunogenicity, and (if applicable) effect of neutralizing antibodies (and cross-reacting antibodies, if applicable) on PK, PD, efficacy, safety	PK, PD, (confirmatory PK/PD), efficacy, safety
Extrapolation	Sufficient scientific evidence for extrapolation must be supported by totality of evidence*	Sufficient scientific justification for extrapolation of clinical data required	Demonstration of non-inferiority to reference product might not support extrapolation to other indications, particularly if other indications include different dosages than those tested	Possible, if sensitive clinical test model used, clinically relevant mechanism of action and/or same receptor, safety and immunogenicity show no unique issues, and non-inferiority demonstrated in efficacy trial
Labeling	Label will copy label of reference product	Likely will include clinical data on both reference product and biosimilar	Cannot entirely duplicate product monograph of reference product; must include statement indicating product is a biosimilar, key data used for Marketing Authorization decision (including tables showing comparisons of biosimilar to reference product), and approved indications, but no claims of bioequivalence or clinical equivalence	May include characterization of and studies performed with biosimilar, but should be as similar to reference product label as possible

* Increasingly, EU regulators see extrapolation as a logical consequence of the comparability exercise principle.^34^

EMA, European Medicines Agency; FDA, US Food and Drug Administration; WHO, World Health Organization; PK, pharmacokinetic; PD, pharmacodynamic

**Figure 1.**
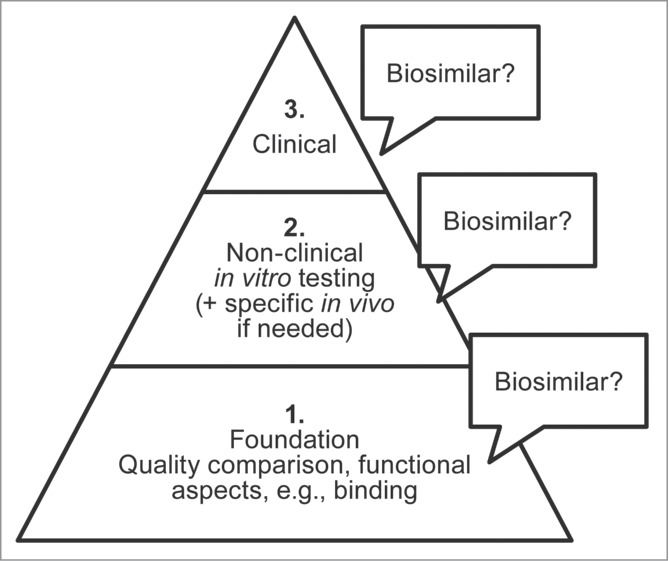
Demonstrating biosimilarity is built first on a foundation of an analytical comparison of structural and functional similarity to the reference product, supported by non-clinical testing, and clinical evaluation in the intended treatment population. Biosimilarity is considered demonstrated based on the *totality of the evidence* from all evaluations, with each step supported by the preceding one.

In order to establish biosimilarity, a stepwise approach is used to develop evidence in support of a potential agent (**Fig. 1**). First, analytical studies are conducted to confirm that the biosimilar has a foundation of quality based on structural and functional similarity to the reference product (notwithstanding minor differences in clinically inactive components). The complete quality dossier required per current legislation includes extensive state-of-the art characterization studies to demonstrate similarity,^12^ which, in practical terms, increases both the size (potentially 2 or 3 times greater in content) and complexity of the analyses required. Next, non-clinical studies need to demonstrate that the biosimilar agent acts on the same target or physiologic process (via assessments of the mechanisms of action and functional activity) and has similar toxicity as the reference product.

A key component of the biosimilarity exercise is a tailored clinical trial program in which PK, clinical efficacy, clinical safety, and immunogenicity of the biosimilar is compared with that of the reference product.^7-10^ The goal of the clinical trial program is not to demonstrate patient benefit, as this has already been demonstrated through the clinical studies conducted for the reference product. Rather, the goal is to show the similarity of the potential biosimilar to the reference product. To accomplish this, clinical trials should be specifically designed to resolve any residual uncertainty as to whether the product can be considered a biosimilar.^7,9^

After non-clinical similarity is established, the first step in clinical evaluation of biosimilarity involves a stepwise procedure that begins with clinical PK comparisons.^7-10^ When appropriate pharmacodynamic (PD) markers exist, PD parameters are usually investigated as a combined PK/PD study.^7-10^ The standard equivalence range used to demonstrate PK bioequivalence for biosimilars generally applies the traditional 80% to 125% equivalence range.^8,10^ A potential biosimilar can be considered comparable to the reference product when the entire confidence interval (CI) falls within the lower and upper limits of this range (i.e., the equivalence margins).^8,10,13^ PF-05280014, a potential biosimilar for trastuzumab, was compared to trastuzumab products marketed in and sourced from the European Union (trastuzumab-EU) and United States (trastuzumab-US) in a recent PK study. The 90% CIs for the ratios of the key PK parameters were all within the bioequivalence window of 80% to 125%. Bioequivalence was also observed between the 2 reference products as trastuzumab-EU to trastuzumab-US were also within this window. These results therefore, support continued development of PF-05280014.^14^

After completion of PK and PD analyses, a clinical study (or studies) to demonstrate that the biosimilar produces comparable efficacy within accepted and predefined limits (the equivalence margin) versus the reference product is conducted.^7-10^ This study also includes evaluation of comparative safety, including immunogenicity assessment, using state of the art methodology and employing appropriate measures of efficacy.

Clinical endpoints employed in clinical trials evaluating biosimilars may be different than those in trials evaluating novel therapeutics because selected endpoints should be sensitive to the detection of product-related differences in efficacy and safety.^15^ For example, although survival is generally a preferred endpoint in many oncology clinical trials, survival endpoints may not be appropriate in clinical trials evaluating biosimilars for oncology indications because of potential confounding due to tumor burden, disease status, or previous lines of therapy.^15-17^ Instead, a measure of response may be a better endpoint for clinical evaluation of a potential biosimilar.^15-17^ The selection of an appropriate response endpoint is complicated by the fact that overall response rate is not always associated with long-term improvements in patient outcome or closely correlate with progression-free survival in patients with metastatic disease.^18-20^ Novel endpoints (such as total pathologic complete response that is associated with disease-free survival in patients with early-stage breast cancer^20^) may add supportive evidence for biosimilarity agents and allow for clinical testing in a sensitive and homogenous population, so their inclusion (especially on an exploratory basis) in clinical trials may be useful in establishing comparable efficacy and safety of biosimilars in oncology indications.^15^

Similar to the clinical PK assessments, evaluation of clinical efficacy and safety in a potential biosimilar is also generally evaluated using equivalence trials.^7-10^^,^^21^ Thus, for 2 therapies to be considered comparable, the same outcome measure for the 2 interventions must fall within a specified range with respect to predefined clinical criteria.^22^ In these trials, equivalence is usually assessed using 2-sided CIs (typically at the 90% or 95% level) for the difference between treatments.^13^ Occasionally, non-inferiority designs, in which the aim is to determine if a therapy is no worse than a standard therapy,^22^ may be considered for biosimilar approvals on an exceptional basis, but the guidelines established by the EMA, Health Canada, WHO, and the FDA draft guidelines prefer the use of equivalence designs for comparison of the proposed biosimilar to the reference product.^7-10^

In order to conduct appropriate analyses of biosimilar products, the equivalence margins used for the main efficacy endpoint must be justified on historical clinical and statistical grounds, and calculated based on the effect size (including consideration of the magnitude and variability of the effect size for the reference product as derived from historic trials).^7-10^^,^^21^ The use of historical data for establishment of appropriate equivalence margins makes the use of novel pathologic endpoints difficult, but (as noted) could provide supportive efficacy and safety data if used on an exploratory basis. One difficulty in calculating the effect size for the reference product is that most of the data available for this calculation will be from randomized clinical trials. These studies often vary widely in trial design and study population, creating yet another challenge for biosimilar developers, particularly for oncology therapies since these studies can often have small sample sizes and widely varying background-chemotherapy regimens.

Across all phases of the clinical trial program, testing must be done in a sensitive and homogenous patient population so that any differences between the biosimilar and the reference product can be easily detected. For PK evaluations, the study population is often healthy subjects because these individuals lack comorbidity and co-medication concerns. However, results from healthy subjects may not reflect PK parameters in the patient population (e.g., when healthy subjects have reduced target-mediated clearance compared with patients); therefore, PK studies are not always possible or feasible in healthy subjects.^7,8^ For the confirmatory efficacy and safety trials, study populations representative of approved therapeutic indications of the reference product and sensitive for detecting potential differences between the biosimilar and the reference product should be employed.^7^

Practical considerations for the selection of an appropriate study population for a single, global Phase 3 study include consideration of the approval status of the reference product for a given indication, as well as the potential for recruiting patients in different countries. Multiple possible choices may exist; therefore, study sponsors must weigh the pros and cons of their choice of specific study populations. In addition, regulatory agencies must agree that a given study population is appropriate to support the application for biosimilar approval. However, the challenges associated with selection of an appropriate study population do not end with agreement with regulatory agencies, since the concept of biosimilarity and the approach to development are novel. As a result, ethics committees not familiar with the concept may raise objections to study designs or selection of study populations based on a misunderstanding of the goals and definitions established to demonstrate biosimilarity. Further education of all stakeholders on this novel regulatory paradigm is necessary.

In summary, although the regulatory paradigm for biosimilars is complex and highly specific, it is abbreviated compared with that of a novel biologic agent. There are several important implications in applying this paradigm to labeling and post-approval safety follow-up. In addition, a number of factors may curtail the uptake of biosimilars and limit patient access to these products.

## Labeling Considerations for Clinicians

Consistent policies in product labeling and prescribing information do not exist across the regulatory agencies. WHO guidelines state that the prescribing information label for biosimilars should be as similar as possible to that of the reference product (particularly for posology and safety-related information, including contraindications, warnings, and adverse events) except for product-specific aspects, such as different excipient(s).^10^ According to WHO guidelines, biosimilar labels potentially may differ from that of the reference product in 2 ways. First, if the biosimilar has fewer indications than the reference product, text related to those indications may be omitted unless information about certain risks is necessary.^10^ If this information is omitted, the prescribing information should clearly state that the biosimilar is not indicated for use in the specific indication(s) and include the reasons why.^10^ Second, a national regulatory authority may choose to mention that the product is a biosimilar, the specific studies that have been performed with the biosimilar, or include specific instructions for the physician on biosimilar product use.^10^

Health Canada has taken a similar approach to the recommendations of WHO. Its guidelines for biosimilar products indicate that the product sponsor will not be allowed to entirely duplicate the product monograph of the reference product for the biosimilar.^8^ Instead, a product monograph for a biosimilar must include a statement indicating that the product is a biosimilar, key data on which the decision for marketing authorization was made, including tables showing the results of the comparisons between the biosimilar and reference product, and the approved indications for the biosimilar, but make no claims of bioequivalence or clinical equivalence between the biosimilar and the reference product.^8^

In contrast, EMA, while agreeing that a statement that the product is a biosimilar is included in the label, has indicated that the labeling of biosimilars follows the same principles used for labeling of small-molecule generics and that the label will copy the label of the reference product.^23^ Specifically, the summary of product characteristics (SmPC) for the biosimilar will be an exact duplicate of the reference product's SmPC; any proposed differences in the biosimilar label need justification.^23^ Recently, the EMA approved the biosimilar infliximab products, Remsima™ (Celltrion Healthcare Hungary Kft., Budapest, Hungary)^24^ and Inflectra™ (Hospira UK Limited, Warwickshire, UK).^25^ The SmPCs for these products duplicate the reference product information with no mention of any clinical trials that compared the biosimilar with the reference product. This means that the evidence used to support approval of the biosimilar, including head-to-head comparisons vs. the reference product, is not provided in the label.^26^ These data may be located in the European Public Assessment Report (EPAR)^27^; however, as they are not mentioned at all in the label, the physician may not know that such studies were conducted and are contained in the EPAR. Thus, there is a lack of ready access to key information in the SmPCs that is the primary reference document for physicians.^11^ The physician may also incorrectly assume that all the clinical data in the label had been generated with the biosimilar product since it is not clear that all data relate to the reference product. Some industry trade associations have highlighted these issues. The European Biopharmaceutical Enterprises (EBE) issued a position paper on the labeling of biosimilars in August 2013.^26^ More recently, EuropaBio issued a similar statement calling for a transparent approach to labeling and inclusion of data on both the reference product and the biosimilar.^28^

To date, the FDA has not issued specific guidance regarding labeling of biosimilars, although the draft guidance for biosimilars states that product labels of a proposed biosimilar product should include all the information necessary for a health professional to make prescribing decisions.^9^ This information should include a clear statement advising that the product is approved as a biosimilar, the specific indications and route of administration, and whether the biosimilar has or has not been determined to be interchangeable (and therefore an option for automatic substitution) with the reference product.^9^

## Post-Approval Considerations

All approved drugs require ongoing safety monitoring.^29^ In addition, more proactive measures to evaluate long-term safety (such as participation in registries with ongoing evaluation of safety in the clinical setting) are often employed. These long-term, proactive plans are designed to understand the nature and frequency of adverse events and possible identification of risk factors.

Specific requirements for post-approval monitoring vary across regulatory agencies, but these plans are required to be discussed with regulatory authorities or submitted as part of the regulatory approval application.^7-10^ For example, the EMA requires a description of the pharmacovigilance system and a risk management plan as part of the regulatory submission.^7^ Similarly, WHO recommends a specific safety and pharmacovigilance plan at the time of submission of the marketing authorization application.^10^ Health Canada requires the risk management plan be presented prior to the issuance of marketing authorization.^8^ Although a position and requirement for pharmacovigilance for biosimilars has not yet been specifically defined by the FDA, they note that existing FDA pharmacovigilance guidelines will likely be considered appropriate.^30^ In the current draft guidance for biosimilars, the FDA encourages discussion with the appropriate regulatory divisions since many aspects of safety monitoring are product-specific.^9^

In general, the regulatory agencies recommend that pharmacovigilance plans should consider any known safety signals associated with the use of the reference product and its class.^7-10^ If any additional specific safety monitoring or pharmacovigilance measures are required for the reference biologic or its product class, a biosimilar should apply the same monitoring/pharmacovigilance plan.^7,10^ In addition, if any novel safety concerns have arisen during evaluation of the biosimilar, these also may be evaluated.^7,10^ Even though the development plan is expected to include an evaluation of immunogenicity as part of the safety assessment of the biosimilar, as for a novel biologic at the time of regulatory approval, the size of the population evaluated and the duration of exposure are likely not large enough to identify rare, but potentially serious safety events, including immunogenicity. As a result, the regulatory agencies specifically indicate that the on-going post-approval follow-up should specifically monitor immunogenicity.^7-10^

## Potential Limitations to the Uptake of Biosimilars

Potential biosimilars are currently in development for oncology indications; however, to date, published data from clinical trials are available for only some of these products (**Table 2**).

**Table 2.** Biosimilar products in development for oncology indications with publications

Reference Product	Proposed Biosimilar (Mfg)	Development Phase	Published Data
Bevacizumab	BCD-021 (CJSC BIOCAD)	3	Phase 3: PK and safety with paclitaxel and carboplatin similar to bevacizumab with paclitaxel and carboplatin^38^
	PF-06439535 (Pfizer)	1	Non-clinical: similar structure and dose-response activity as bevacizumab sourced from US and EU^39,40^
Rituximab	BCD-020 (CJSC BIOCAD)	3	Phase 3: equivalent PK with comparable PD and safety to rituximab^41^
	GP2013 (Sandoz/Novartis)	3	Non-clinical: physicochemical and functional characterization similar to rituximab sourced from US and EU^42^; in vivo comparability in PK, PD, and efficacy to rituximab^43^
	PF-05280586 (Pfizer)	3	Phase 1: comparable PK to rituximab sourced from US and EU^44^; comparative PD, safety, tolerability, and immunogenicity to rituximab sourced from US and EU in patients with rheumatoid arthritis^45^
	RTXM83 (mAbxience)	3	Non-clinical: similar structural attributes, purity, binding affinity; in vitro and in vivo potency; in vivo PK and PD to rituximab sourced from EU^46^
	CT-P10 (Celltrion)	1	Phase 1: comparable PK and safety to rituximab sourced from EU^47,48^
Trastuzumab	BCD-022 (CJSC BIOCAD)	3	Phase 3: PK and safety after administration with paclitaxel; similar to trastuzumab with paclitaxel in patients with HER2+ MBC^49^
	CT-P6 (Celltrion)	3	Phase 1/2b: equivalent PK and similar safety to trastuzumab in patients with HER2+ MBC^50^ Phase 3: equivalence for ORR in patients with HER2+ MBC in combination with paclitaxel, comparable efficacy in other endpoints, safety profile comparable to trastuzumab^51^
	PF-05280014 (Pfizer)	3^52,53^	Non-clinical: similar structure, function, chromatographic profile, PK, and immunogenicity to trastuzumab sourced from US and EU^54^ Phase 1: comparative PK, PD, safety, tolerability, and immunogenicity to trastuzumab sourced from US and EU, in healthy subjects^14^

Mfg, manufacturer; PK, pharmacokinetics; PD, pharmacodynamics; HER2, human epidermal growth factor receptor 2; ORR, overall response rate; MBC, metastatic breast cancer

Despite biosimilars providing additional treatment options and having demonstrated similarity to the reference product, it is likely that physicians will not consider biosimilars to be interchangeable with the reference product and, therefore, should not be automatically substituted (i.e., substituted at pharmacy level without a physician's consent). The EMA does not have the legal remit to determine interchangeability; these decisions are determined by policies adopted by national member states. Some of these individual regulatory agencies have enacted policies that prohibit automatic substitution. Health Canada is deferring this decision to the provinces and territories.^8^ In contrast, the FDA has the legal authority to determine whether a product approved as a biosimilar may attain the higher level of evidence required for approval as an interchangeable biosimilar, but individual state laws will also need to be applied.^9^ To date, guidance has not yet been issued by the FDA on interchangeability.^9^

Another key concept in the regulation of biosimilars is the possibility to extrapolate indications, i.e., allowing approval of a biosimilar for use in indications of the reference product even if the biosimilar has not been evaluated in a clinical trial in that specific indication.^10^ A common misconception about extrapolation is that the focus is on the clinical data alone for making the justification.^31^ However, since the clinical evidence is only part of the data supporting the regulatory application for a biosimilar, the justification for extrapolation is based on the overall totality of data generated on similarity of the biosimilar to the reference product, including non-clinical data such as analytical and in vitro functional comparisons and the mechanism of action in the indications concerned. Evaluation of a biosimilar for extrapolation to additional indications is conducted on a case-by-case basis and depends on the level of evidence provided by the applicant. Extrapolation of indications is a complex area and, not surprisingly, regulatory agencies are coming to different conclusions on the extent of extrapolation allowed. For example, EMA recently granted approval of the full range of indications of the reference product to the biosimilar infliximab products Remsima^24^ and Inflectra,^25^ whereas Health Canada determined that, because of functional differences between the biosimilar and the reference product, which were also correlated with pertinent differences in the mechanism of action, the data provided did not support extrapolation to Crohn's disease and ulcerative colitis.^32,33^ A further complicating factor when considering extrapolation is that every biosimilar is a version of the target reference product. Not all biosimilar versions of the same target reference product are the same and therefore, the data presented for each individual product will not be the same. Therefore, regulators need to make case-by-case assessments on the merits of extrapolation. There is no one-size-fits-all approach. The nuanced nature of this assessment may make physician acceptance of this concept more challenging. However, it is important to recognize that the possibility of extrapolating indications is very important for the development of biosimilars because, without this option, the biosimilar developer would have to repeat the entire clinical development program of the reference product to gain approval in all indications. This is contrary to the intent of developing biosimilars and, if this was necessary, development of a potential biosimilar would be more extensive, likely leading to increased development costs and time and potentially reduced patient access to the biosimilar.

Similar to novel biologic therapies, the long-term safety of biosimilars will be limited at the time of approval. As discussed, post-approval safety monitoring will be required as part of pharmacovigilance requirements. These requirements are similar to those required for any approved biologic agent and can often limit initial use of a novel therapy. Because clinicians will initially be unfamiliar with biosimilars as a new type of biological product, improved communication not only to physicians, but also to payers and patients about the labeling and the rigor of oversight for biosimilars, including post-marketing surveillance, is necessary.^34^

## Conclusions

Despite the complex developmental and regulatory processes involved, development of biosimilars to treat cancer should provide additional treatment options that have the potential to generate savings and efficiencies for healthcare systems, which can help free up resources for other healthcare treatments and interventions.^4,35,36^ This is due, in part, to the projected savings in commercial development associated with the abbreviated approval process for biosimilars compared with novel biologic therapies.^37^ Biosimilars have already improved access to well-established therapeutic interventions in Europe and other locations where approvals have occurred.^4,35^

Although there are some limitations to the use of biosimilars due to issues with interchangeability and extrapolation of data, in the long-term, biosimilars should offer a wider array of therapeutic solutions and increase accessibility to effective cancer treatments. Clinicians should understand the importance of biosimilars in clinical practice and how to make informed decisions about their appropriate use. In countries where biosimilars have been approved by regulatory agencies, changes in the practice of medicine have already occurred.^4^ The on-going development of additional biosimilars should produce further improvements in the care and treatments of patient with cancer due to expanded use of biologic therapies, which may lead to better overall health outcomes and changes in medical guidelines and treatments, allowing more patients access to effective cancer treatments.
